# An Ethical Point

**Published:** 1911-07-01

**Authors:** J. A. Morgan


### INSTITUTIONAL LETTER-BOX.

### AN ETHICAL POINT.

*To the Editor of* The Hospital.

Dear Sir,—As a subscriber to The Hospital, I should like your opinion, either publicly or privately, on the enclosed copy of a typewritten letter as to whether it is in strict accord with medical ethics.

The autograph letter I retain, but it is open for inspection to those who desire to see it.

For personal reasons, the doctor who received it removed his name and the date.—Yours faithfully,

J. A. Morgan.

[The copy of the typewritten letter referred to certainly seems to transgress the spirit of medical ethics, because it oversteps the boundaries of ordinary neighbourliness and gentlemanly conduct. So long as communications of this sort are limited to members of the profession, probably little harm is done. The most objectionable feature is, of course, the implication that the writer's mineral spring and his treatment are superior to those which can be obtained at the neighbouring spa. Dr. Morgan seems entitled to sympathy, for the letter is clearly an attempt to attract away his clients. We doubt, however, whether the letter in question, being one addressed privately to another medical man, could be described as infamous conduct in a professional sense. We should recommend Dr. Morgan to consult the opinion of his confrères at the local branch of the British Medical Association (if he belongs to it); or to take the advice of the oldest and most respected practitioner within twenty miles; or to do nothing at all, confident that a rival who has to resort to such methods is one from whom he has little to fear.—Ed. The Hospital.]

